# The 24-hour cognitive-affective physical behavior model: a theoretical framework for studying determinants and health consequences of physical activity, sedentary behavior, and sleep

**DOI:** 10.1186/s44167-025-00077-9

**Published:** 2025-04-08

**Authors:** Marco Giurgiu, Ulrich W. Ebner-Priemer

**Affiliations:** 1 Institute of Sports and Sports Science, Karlsruhe Institute of Technology (KIT), Engler-Bunte- Ring 15, 76131 Karlsruhe, Germany; 2 Department of Psychiatry and Psychotherapy, Central Institute of Mental Health, Medical Faculty Mannheim, University of Heidelberg, Mannheim, Germany

**Keywords:** Physical behavior, Theoretical framework, Determinants, Consequences, Executive function, Affect, Fitness, Health

## Abstract

Physical behaviors (i.e., sleep, sedentary behavior, and physical activity) are major drivers of physical and mental health. Existing behavior change models (e.g., social-cognitive models, humanistic/organismic models, socioecological models, or dual-process models) only account for a modest proportion of the differences in physical behavior between and within individuals. This limitation may stem from three key shortcomings. First, these models tend to focus either on short-term determinants or long-term consequences. Second, they overlook the bidirectional nature of many of these relationships, where consequences can also function as determinants. Third, physical activity, sedentary behavior, and sleep are intricately interrelated, constrained by the fixed duration of 1440 min per day. To overcome these limitations, we introduce the *24-hour cognitive-affective physical behavior* model. At the center of this model is the 24-hour physical behavior construct, which is embedded within a framework of determinants and consequences, interconnected by eight partially reciprocal pathways that operate on distinct temporal scales. Our model offers a holistic, multi-behavior perspective, shifting away from single-behavior approaches, and introduces clearly defined temporal dimensions to test associations between 24-hour physical behavior compositions and cognitive-affective determinants as well as health consequences. The long-term objective of this framework is to identify the optimal balance of 24-hour physical behaviors that promotes sustained health and longevity.

**Supplementary Information:**

The online version contains supplementary material available at 10.1186/s44167-025-00077-9.

## Background

Movement and non-movement behaviors also known as time-use behaviors or physical behavior (PB) (i.e., sleep, physical activity (PA), and sedentary behavior (SB)) [1] are crucial for preventing and regulating various health conditions, including chronic diseases such as physical and mental health issues [2–6]. Over the past years, the 24-hour PB perspective has become a central part of conceptual models, approaches, and hypotheses. In particular, Pedišić [7] introduced first the Activity Balance Model, a theoretical framework for investigating associations of 24-hour PB with health outcomes, and a couple of years later together with his team the Framework for Viable Integrative Research in Time-Use Epidemiology [8] as an extension, while incorporating research on methods, outcomes, optimal balance, determinants, and effectiveness of interventions. Rosenberger and colleagues [9] introduced the 24-hour Activity Cycle (24-HAC) model as a paradigm for exploring the inter-relatedness of health effects and PB. Holtermann and colleagues [10] postulate that the “Sweet-Spot” of 24-hour PBs for better health differs between adults, depending on their occupation. Collectively, this body of work provides a strong foundation for the development of our proposed model, *The 24-hour cognitive-affective physical behavior model*. All of the models and frameworks mentioned above focus on the 24-hour PB construct and go beyond the consideration of a single behavior. However, our model, which is described below, differs from the existing work. While the 24-HAC model [9] is a conceptual model that emphasises research purposes (e.g., guideline development) and the work of Holtermann and colleagues [10] is a specific hypothesis rather than a model, our approach includes determinants and consequences of 24-hour PB. Whereas the Activity Balance Model [7] refers to health consequences and points to the relevance of moderators and confounding variables, the model includes no determinants such as behavioral factors. This aspect is certainly addressed by the Viable Integrative Research in Time-Use Epidemiology framework [8], but at a higher level of abstraction without going down specific paths (e.g., adoption of dual-process mechanisms as determinants). Moreover, the existing frameworks do not specify the temporal resolution, i.e., whether we should expect to see the effects within hours, days, weeks, or months.

Besides the 24-hour PB frameworks, traditional behavior change theories and models have been proposed to either identify determinants of PB or to explore the health consequences of PB, including social-cognitive models (e.g., theory of planned behavior), humanistic/organismic models (e.g., self-determination theory), socioecological models [11] or dual-process approaches (e.g., affective-reflective theory, theory of effect minimization [12, 13]). Especially, cognitive-affective determinants play a crucial role in shaping both health behaviors and physical behaviors. These determinants encompass psychological factors such as beliefs, attitudes, emotions, and motivation, which influence an individual’s decision-making and actions related to their well-being. Understanding how cognitive and affective processes interact can help explain why people adopt or avoid certain health behaviors, including exercise adherence [14]. While these behavior change theories and models have undoubtedly been important in understanding why some people are active while others are not, they only account for a modest proportion of the differences in PB between and within individuals [15, 16]. To increase predictability and explainability, three shortcomings should be resolved. First, existing models often focus exclusively on either the determinants [7, 17] or the consequences of PB [18]. There is a need for integrated models that address both aspects (e.g., as done in the VIRTUE framework [8]), offering a comprehensive understanding of how determinants can be harnessed to promote PB, ultimately enhancing fitness and health outcomes. Second, consequences may appear as determinants for subsequent behavior, as some associations may underlie a reciprocal nature. PB does not only influence fitness and health but good fitness and health does influence the daily life compositions of PB [19, 20]. For example, adequate sleep is essential for recovery, cognitive function, and metabolic regulation [21], while excessive sedentary behavior is linked to adverse health effects such as obesity, cardiovascular disease, and metabolic disorders [5]. Engaging in regular physical activity contributes to improved cardiovascular fitness, muscle strength, mental well-being, and longevity [22]. Most importantly, reciprocal associations may work on a different time scale. While fitness is a long-term outcome of regular exercise, it also serves as a short-term determinant that influences subsequent PB. Thus, reciprocal relationships should be integrated into theoretical models to provide a more accurate representation of the dynamics between PB, determinants, and consequences (e.g., neurocognitive affect-related model [17] or health model [18]). Third, a day is limited to 1440 min. Accordingly, the components of PA, SB, and sleep are extremely linked together in the real world. We cannot increase the time spent in one of the three behaviors limitless, each physically active minute substitute another minute of SB or sleep, which have to be acknowledged in concepts, models and analytical approaches [7–10].

In summary, there is a need for models that integrate behavioral and affective determinants, health consequences, and their interactions into a unified framework that encompasses sleep, SB, and PA. Our short report aims (i) to introduce *the 24-hour cognitive-affective physical behavior model*; (ii) to describe how the 24-hour concept is embedded in our model and linked to behavior change approaches; and (iii) to emphasize the relevance of the temporal resolution when studying associations of the model paths.

### Description of the 24-hour physical behavior model

**Fig. 1:**
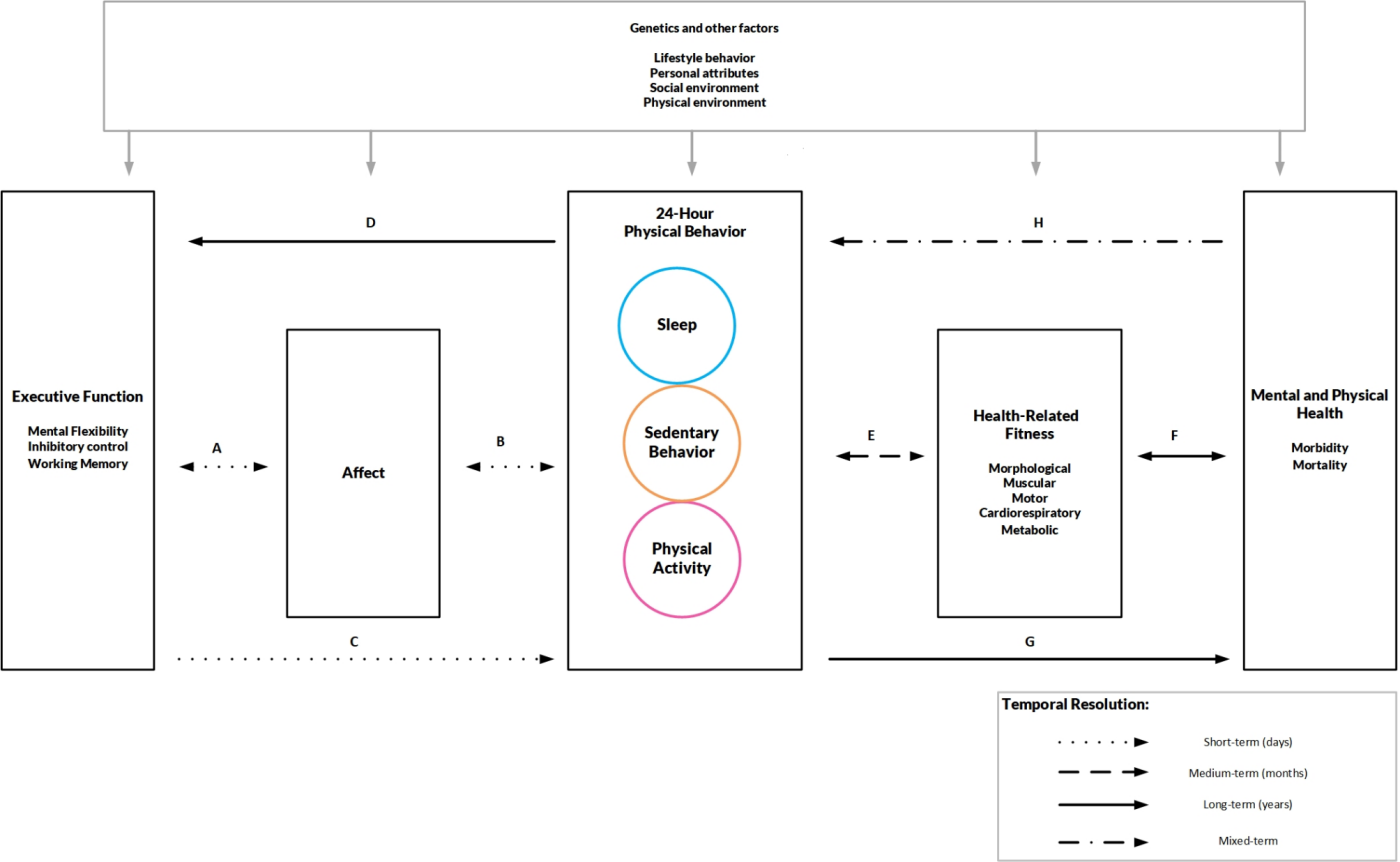
The 24-hour cognitive-affective physical behavior model

The center of *the 24-hour cognitive-affective physical behavior model* (see Fig. 1) represents the 24-hour PB construct, which is surrounded by two sides (left the determinant and right the health outcome side) and eight paths (A to H). The integration of the 24-hour PB construct, based on existing frameworks such as the Activity Balance Model [7], the Framework for Viable Integrative Research in Time-Use Epidemiology [8], or the 24-HAC cycle [9], represents a shift from a single-behavior to a multi-behavior perspective (see the next section for details), while the proposed model includes a number of cognitive, affective and health-related fitness considerations.

The left side with paths A to D is based on the *Neurocognitive Affect-Related Model* [17] and shows an exemplary representation of a dual process approach for the prediction of upcoming 24-hour PB. Central constructs of the paths include the interplay between affective states, executive functions, and compositions of PB. Specifically, following the research of Duncan and Barrett, we view affect as a neurophysiological state characterized by two properties: experiencing feelings of pleasantness or unpleasantness (valence) and levels of activation or deactivation (energetic arousal) [23]. According to Diamond [24], executive functions enable mentally playing with ideas, taking time to think before acting, handling novel and unexpected challenges, resisting temptations, and maintaining focus. The core executive functions include inhibition control (e.g., impulsive actions and interference control), working memory, and cognitive flexibility (e.g., viewing situations from different perspectives) [24]. In summary, optimal compositions of 24-hour PB (i.e., allocation of a fixed period (typically 24 h) across sleep, SB, and PA) may promote increased executive functions (path D), which may help to foster a positive affective response (path A) and may ultimately help to facilitate future compositions of PB (path B). Further, we assume a reciprocal relationship between compositions of PB and executive functions (paths C and D). More specifically, path A suggests that neurocognition, particularly executive function-based cognition, may significantly influence and shape affective responses induced by the compositions of PB (e.g., exercise or sedentary-induced affective responses). Path B connects an individual’s affective response to compositions of PB with their future balance of time spent in PBs. For example, affective responses during exercise are linked to future exercise engagement and affective judgments about subsequent exercise behavior [25]. It is worth noting that pathways A and B may also underlie a bidirectional relationship since studies have shown that PB can also predict momentary affect [26–28] and momentary affect may also predict executive functions [29]. Pathways C and D propose a cyclical, bidirectional relationship within this model. Executive function indirectly influences the compositions of PB through affective responses, while compositions of PB itself may significantly impact cognitive function [30, 31]. For instance, individuals with optimal executive function are generally capable of maintaining a suitable mental state to achieve future goals. This involves planning, filtering out competing information, sustaining efforts despite distractions, and inhibiting responses that are inconsistent with their goals [24, 32].

The right side with the paths E to H is based on the health model presented by Bouchard, Blair, and Haskell [18]. The modification of the model describes the relationships between compositions of PB, health-related fitness, and state of health (i.e., physical and mental health). The core statement of our model supposes that the optimal composition of PB in everyday life improves health-related markers of fitness (path E) and thus also the state of health (e.g., increased well-being or reduced mortality risk) (path F). Several systematic reviews revealed that 24-hour PB compositions are associated with health-related markers and health status [33–37]. For example, Grgic et al. (2023) concluded based on a systematic review of fifty-six studies that time reallocation between sleep, SB, light to moderate physical activity (LPA), and moderate to vigorous physical activity (MVPA) may be associated with a number of health outcomes [33]. Pathways E and F may also underlie a bidirectional relationship since studies have shown that fitness marker can also predict single behaviors of 24-hour PB construct [38, 39] and health status may also predict fitness marker [40, 41]. Notably, the impact of sleep on health should not be neglected [42, 43]. For example, a meta-analysis has shown that short sleep was significantly associated with mortality and various health outcomes such as diabetes mellitus, cardiovascular diseases, coronary heart diseases, and obesity [44].

The presented model focuses on identifying the optimal composition of PB and its effects on fitness markers and overall health. The interaction of these pathways can be summarized using the following working example: One goal might be to achieve balanced sleep, minimize sedentary time, and incorporate sufficient physical activity into daily life. Cognitive preparation (e.g., time management and goal setting) for planning the upcoming day can influence PB compositions (Path C). Previous affective responses to 24-hour PB also play a significant role in this cognitive preparation (e.g., a positive affective response to an evening jogging session) (Path A). Additionally, moment-to-moment fluctuations in affective states can directly influence upcoming PB (e.g., sunshine could enhance affective state and encourage outdoor jogging activity) (Path B). The 24-hour PB compositions at the end of the day subsequently influence future cognitive functions (e.g., regular exercise improving executive functions) (Path D). These cognitive-affective determinants shape the 24-hour PB and result in effects on fitness markers and health. For instance, increased time spent exercising within the 24-hour PB composition can enhance health-related fitness markers (e.g., cardiorespiratory fitness) (Path E), ultimately affecting both physical and mental health (Paths F and G). Conversely, chronic illnesses, respiratory infections, or sports and recreational injuries can directly impact the 24-hour PB compositions (e.g., a broken leg prevents from being physically active and thus, increasing SB and sleep) (Path H). Drawing on a recently published umbrella review [32] and additional empirical studies, we present a summary of the evidence for the pathways in the supplement (see Supplementary File 1).

It is important to note that the relationships between PB composition, health-related fitness markers, and overall health status may be more complex than described. These relationships are influenced or shaped by factors such as genetic predispositions and life circumstances, including social and physical environments as well as individual characteristics. It is worth noting that these factors may play different roles in the pathways (e.g., moderation, mediation, confounding) or regarding the central constructs. Accordingly, generalizing these relationships between 24-hour PB composition, health-related fitness markers, and overall health status is challenging due to the variability in individual circumstances and genetic factors. As a simple example, even the regular performance of PA is dependent on different determinants and illustrates the complexity in this context [45]. Furthermore, mediation analyses may occur between the central components of the model (i.e., executive function, affect, 24-hour PB, health-related fitness, and health status) [46]. For instance, 24-hour PB compositions could influence affect, which, in turn, may predict health-related outcomes.

### Extension to the 24-hour physical behavior concept

After 70 years and thousands of publications, the field of PA research can be characterized by improving methodologies and shifting paradigms. For example, device-based measures via wearables have become increasingly common in PB research and have offered unprecedented opportunities to study PB in granularity with much better accuracy [47]. Today, it is possible to capture all facets of the 24-hour PB construct within a single day using time-stamped data [48]. The 24-hour perspective is an important step towards a holistic view of everyday PB and their impact on health (path G). Here, the previously mentioned frameworks (i.e., the Activity Balance Model [7], the Framework for Viable Integrative Research in Time-Use Epidemiology [8], or 24-HAC model [9]) provide an essential work that highlights the significance of the shift from focusing on single behaviors to adopting a holistic approach. Tremblay and colleagues expect that in the future, the 24-hour approach will provide a platform for individualized precision 24-hour movement behavior guidelines that customize recommendations to individual characteristics and circumstances making them more tailored and equitable [49]. Several countries as well as the WHO provide 24-hour movement guidelines [50–56]. The potential usefulness of this approach needs analytical and statistical ways of understanding the temporal patterning of 24-hour PB. A scoping review by Leech and colleagues aimed to identify novel analytic methods for determining temporal behavior patterns and their contextual correlates [57]. Next to machine learning approaches with various clustering algorithms and model-based clustering techniques, researchers recently used compositional data analysis (CoDA). By applying CoDA, PBs within the 24-hour framework are analyzed relative to each other, rather than as individual entities [58]. In addition, the compositional isotemporal substitution method presents a way to estimate change in a health outcome when fixed durations of time are reallocated from one part of a particular time-use composition to another, while the remaining parts are kept constant [59]. Further, a recently published article by von Rosen [60] presents and discusses four different approaches to analyzing the composition of time use as a dependent variable, which is suitable for analyzing paths B, C, E or, H in our model.

The integration of the 24-hour PB construct as the center of the model with the statistical application of time-use data can be seen as a promising step towards a more holistic view. Notably, we integrated the 24-hour PB construct, distinguishing it into three compositional components: sleep, PA, and SB. However, the number of components can be further refined based on a specific research question. For instance, PA can be subdivided into standing, LPA, and MVPA, while sleep can be categorized into different stages, such as rapid eye movement (REM) and non-REM. We anticipate that future research endeavors might be also interested in context-specific compositions, such as time spent being physically active in outdoor versus indoor environments. Research over the last decade has shown that studies applying CoDA added value in terms of the understanding of health effects thus underpinning our approach for the extension from a single to a compositional perspective. For example, Blodgett and colleagues [61] analyzed data from six cross-sectional studies with a sample of 15.253 participants. Compositions of PB were built on device-measured time in sleep, SB, standing, LPA, and MVPA. CoDA analyses revealed that MVPA has the strongest, most time-efficient protective associations with cardiometabolic outcomes. Further, SB was the sole behavior with clear adverse associations with outcomes, regardless of duration [61]. Associations of sitting, standing, PA, and sleep with cardiometabolic health and glycaemic control markers were studied in The Maastricht Study with a sample of 2.388 participants. Brakenridge and colleagues concluded that shorter sitting time and more time spent standing, undergoing PA, and sleeping are associated with preferable cardiometabolic health [62] (see also Supplement File 1).

### Relevance of temporal resolution

As an extension to previous models, *the 24-hour cognitive-affective physical behavior model* addresses the temporal resolution of the paths, which directly impacts methodological considerations such as the selection of assessment tools, the study design, and the analytical approach. Notably, the following examples are merely one possible way and should not be understood as an exclusive option. The intention is to enable future study results to be incorporated into the model while differentiating the perspective of temporal resolution. In simple words, to study and model the paths, time is a central indicator [63]. We differentiate the eight paths (A to G) into short (within days), medium (across weeks and months), long (over years), and mixed temporal resolution (somewhat between days and years).

Inspired by complex dynamic system models [64], we describe paths A, B, and C as dynamic, reflecting short-term associations between the constructs of interest. This dynamic behavior is particularly evident in affective states, which typically fluctuate throughout the day, from hour to hour or even minute to minute [65]. Moreover, technological innovations, such as mobile cognitive assessments [66]), have prompted researchers to reconsider the conventional view of executive functions as stable traits. Recent studies emphasize the importance of accounting for within-person temporal variability [67]. To capture these short-term fluctuations and understand the dynamic associations between executive functions, affect, and 24-hour PB over time, high-granularity data is essential. Ambulatory assessments (AA) [68, 69], which combine real-time self-reports via smartphone-based electronic diaries with device-based measures of 24-hour PB from wearables, may facilitate this process [70]. Leveraging the methodological advancements of mobile and electronic devices presents a viable approach to capturing short-term fluctuations just in time under real-world conditions [71, 72]. This AA approach represents the state-of‐the‐art methodology for examining dynamic within‐person associations in everyday life. It utilizes real‐time, objective, device‐based methods with repeated measurements and a high sampling frequency while enhancing ecological validity and reducing traditional retrospective biases [68, 73, 74].

The medium temporal paths (E) describe associations over a time frame of several weeks and months. Compared to short-term paths with daily analyses, associations between 24-hour PB and markers of health-related fitness underlie physiological mechanisms over longer periods. Therefore, to unveil associations between 24-hour PB and markers of health-related fitness, longer observation periods are required. In this context, most of previous studies conducted intervention studies. Optimally, the study design includes randomized groups with repeated measurements as well as follow-up measurements and the differentiation of within- and between-person effects.

The long temporal paths (F, G) describe associations over a longer time frame such as years. Based on empirical evidence, health benefits from a good level of fitness may develop over a longer period. For example, a meta-analysis by Hespanhol Junior revealed that after a year of training with approximately three sessions per week several health markers such as body mass, body fat, and resting heart rate were improved [75]. Long-term cohort studies could provide a unique insight into the interaction between fitness and health over the years. A community-based longitudinal study over 18 years reported that habitual PA showed a positive relationship with physical fitness and health status [76].

Finally, the mixed temporal path (H) describes associations with a variation between days and years. For instance, prediction of upcoming 24-hour PB can be influenced by daily health status such as respiratory infection, long-term injuries or diseases that change the compositions of PB over a short or longer period [77]. Here, the temporal resolution of the path depends on the specific research question. Finally, it should be noted that the PB construct is inherently tied to the fixed duration of 24-hour per day, which implies observations and analyses on a daily level. In line with findings that past behavior can influence future behavior [78], we suggest that past 24-hour PB should also be incorporated into within-subject analyses for predicting future 24-hour PB.

## Conclusion

The presented *24-hour cognitive-affective physical behavior model* synthesizes existing theoretical considerations [17, 18], providing both, a more holistic view moving from a single to a multi-behavioral perspective and specific paths with defined temporal resolution to test associations between 24-hour PB compositions and health consequences as well as determinants. It follows existing models [7–9] in the way that it integrates the 24-hour PB approach, extending beyond a single behavior perspective. Further, it extends existing models while it combines behavioral, affective, and health state determinants and consequences of 24-hour PB and it emphasizes the importance of temporal resolution and its impact on methodological aspects. Our model should be recognized as a flexible framework that can be individually adapted. A key long-term objective of this model is to predict the optimal balance of 24-hour PB that maximizes health benefits and promotes longevity and well-being.

## Electronic supplementary material

Below is the link to the electronic supplementary material.

Supplementary Material 1

## Data Availability

No datasets were generated or analysed during the current study.

## Abbreviations

PB: Physical behavior
PA: Physical activity
LPA: Light physical activity
MVPA: Moderate-to-vigorous physical activity
SB: Sedentary behavior
CoDA: Compositional data analysis
