# Supplementary material for: The 24-hour cognitive-affective physical behavior model: a theoretical framework for studying determinants and health consequences of physical activity, sedentary behavior, and sleep
Source: J Act Sedentary Sleep Behav. 2025 Apr 8;4:6. doi: 10.1186/s44167-025-00077-9 (PMC11977878; doi:10.1186/s44167-025-00077-9)
Supplement: Supplementary file 1 — Supplementary Material 1 [file 44167_2025_77_MOESM1_ESM.docx]

Supplement 1: Selected studies that indicate the empirical evidence of the paths.

| **Path** | | **Conclusion** |
| --- | --- | --- |
| A | Executive Function ↔ Affect | - Individuals differed in how reliably working memory performance fluctuated across days, and that subjective experiences (e.g., higher levels of negative affect) were primarily linked to performance accuracy (1). - Psychological resilience moderated the relationship between momentary inhibitory control and momentary mood (2). - Negative moods and not positive moods modulated both the short and long time scales of cognitive flexibility but with dissociable effects (3). |
| B | Affect ↔ 24-hour PB | - Already short physical activity bouts in everyday life, which clearly differ from structured exercise sessions, are positively associated with affective well-being (4). - Longer sleep duration at the expense of time awake in bed was associated with lower high arousal negative afect. More moderate-to-vigorous physical activity (MVPA) at the expense of light-physical activity (LPA) or sedentaray behavior (SB) was associated with higher high arousal positive afect (5). - Higher ratio of the SB to LPA and MVPA composition within the 60 min prior to a diary rating negatively influenced valence and energetic arousal. A higher ratio of LPA to SB and MVPA positively influenced energetic arousal and a higher ratio of MVPA to SB and LPA positively influenced valence and energetic arousal (6). |
|  |  |  |
| C | Executive Function 🡪 24-hour PB | - Executive functions might be a relevant predictor of physical activity with a small but significant total effect size (7). - Greater executive function is associated with less follow-up sedentary behavior (8). - Decrements in selected executive function scales are associated with subjective sleepiness, but not sleep duration, in adolescents (9). |
| D | 24-hour PB 🡪 Executive Function | - The 24-hr composition was positively associated with executive functions, and that reallocating 5, 10, 15 or 20 min of the time spent on sleep and light PA to MVPA, respectively, was associated with significant improvements in executive function (10). - Spending more time in MVPA was associated with better long-term memory scores, but only for those with smaller frontal lobe volume. Conversely, spending more time in sleep and less time in sedentary behaviour was associated with better executive function in those with smaller total grey matter volume (11). - LPA was positively associated with inhibitory control, and this association was stronger in bouts of LPA than in sporadic LPA. Moreover, reducing the time spent in SB or sleep and increasing the time spent in LPA, especially long-bout LPA, could be important measures for managing inhibitory control in late life (12). |
| E | 24-hour PB ↔ Health-related Fitness | - Meeting sleep, physical activity and sedentary behavior guidelines was favorably associated with adiposity, cardiometabolic health, mental and social health, physical fitness, health-related quality of life, academic achievement, cognitive development, perceived health, dietary patterns, and myopia (13). - Findings underscore the critical role of increasing MVPA and reducing SB in improving cardiorespiratory fitness among the older adult (14). - A bidirectional relationship may exist between metabolic diseases such as type 2 dibates mellitus and obstructive sleep apnea (15) |
| F | Health-related Fitness ↔ Mental and Physical Health | - Cardiorespiratory fitness levels are associated with total and abdominal adiposity. Further, cardiorespiratory fitness have short-term and long-term positive effects on depression, anxiety, mood status and self-esteem in young people (16). - There is a longitudinal association between cardiorespiratory fitness levels and the risk of a common mental health disorder (17). - Children with chronic diseases had reduced physical activity and cardiorespiratory fitness (18). |
| G | 24-hour PB 🡪 Mental and Physical health | - Low physical activity and short sleep, low physical activity and mid sleep, high physical activity and long sleep, and low physical activity and long sleep were associated with risk of all-cause mortality (19). - Reductions in mortality risk are greater when time spent sedentary is replaced with higher intensities of physical activity (20). - Time displacement estimates revealed that the greatest estimated changes in mortality risk occurred when time spent in MVPA was decreased and replaced with sleep, SB, LPA or a combination of these behaviors (21). |
| H | Mental & physical health 🡪 24-hour PB | - Low physical activity levels and high levels of sedentary behaviors were found consistently after injury (22). - Poorer sleep efficiency and shorter sleep duration in the weeks preceding exposure to a rhinovirus were associated with lower resistance to illness (23). - People with depression are at increased risk of engaging in high levels of SB than non-depressed participants (24). |

**References:**

1. Brose A, Schmiedek F, Lövdén M, Lindenberger U. Daily variability in working memory is coupled with negative affect: the role of attention and motivation. Emotion (Washington, DC). 2012;12(3):605–17.

2. Nahum M, Sinvani RT, Afek A, Ben Avraham R, Jordan JT, Ben Shachar MS, u. a. Inhibitory control and mood in relation to psychological resilience: an ecological momentary assessment study. Sci Rep. 12. August 2023;13(1):13151.

3. Hsieh S, Lin SJ. The Dissociable Effects of Induced Positive and Negative Moods on Cognitive Flexibility. Sci Rep. 4. Februar 2019;9(1):1126.

4. Timm I, Giurgiu M, Ebner-Priemer U, Reichert M. The Within-Subject Association of Physical Behavior and Affective Well-Being in Everyday Life: A Systematic Literature Review. Sports Med [Internet]. 6. Mai 2024 [zitiert 17. Mai 2024]; Verfügbar unter: https://link.springer.com/10.1007/s40279-024-02016-1

5. Le F, Yap Y, Tung NYC, Bei B, Wiley JF. The Associations Between Daily Activities and Affect: a Compositional Isotemporal Substitution Analysis. International journal of behavioral medicine. 2021;

6. Giurgiu M, Ebner-Priemer UW, Dumuid D. Compositional insights on the association between physical activity and sedentary behavior on momentary mood in daily life. Psychology of sport and exercise. 2022;58:102102.

7. Gürdere C, Strobach T, Pastore M, Pfeffer I. Do executive functions predict physical activity behavior? A meta-analysis. BMC Psychol. 2. Februar 2023;11(1):33.

8. Loprinzi PD, Nooe A. Executive function influences sedentary behavior: A longitudinal study. Health Promot Perspect. 1. Oktober 2016;6(4):180–4.

9. Anderson B, Storfer-Isser A, Taylor HG, Rosen CL, Redline S. Associations of Executive Function With Sleepiness and Sleep Duration in Adolescents. Pediatrics. 2009;123(4):e701–7.

10. Bezerra TA, Clark CCT, Souza Filho AND, Fortes LDS, Mota JAPS, Duncan MJ, u. a. 24‐hour movement behaviour and executive function in preschoolers: A compositional and isotemporal reallocation analysis. European Journal of Sport Science. Juli 2021;21(7):1064–72.

11. Mellow ML, Dumuid D, Olds T, Stanford T, Dorrian J, Wade AT, u. a. Cross-sectional associations between 24-hour time-use composition, grey matter volume and cognitive function in healthy older adults. Int J Behav Nutr Phys Act. 30. Januar 2024;21(1):11.

12. Hyodo K, Kitano N, Ueno A, Yamaguchi D, Watanabe Y, Noda T, u. a. Association between intensity or accumulating pattern of physical activity and executive function in community-dwelling older adults: A cross-sectional study with compositional data analysis. Front Hum Neurosci. 25. Januar 2023;16:1018087.

13. Zhao H, Wu N, Haapala EA, Gao Y. Association between meeting 24-h movement guidelines and health in children and adolescents aged 5–17 years: a systematic review and meta-analysis. Front Public Health. 7. Mai 2024;12:1351972.

14. Lu D, Zhang W, Tan S. Assessing the impact of 24-hour activity behaviors on cardiorespiratory fitness in the older adult: a component analysis approach. Front Public Health. 18. November 2024;12:1478533.

15. Patil S, Tak S, Mirza AW. Diabetes mellitus, metabolic syndrome, and sleep disorders: An underestimated relationship. Ann Med Sci Res. Mai 2024;3(2):91–101.

16. Ortega FB, Ruiz JR, Castillo MJ, Sjöström M. Physical fitness in childhood and adolescence: a powerful marker of health. Int J Obes. Januar 2008;32(1):1–11.

17. Kandola A, Ashdown-Franks G, Stubbs B, Osborn DPJ, Hayes JF. The association between cardiorespiratory fitness and the incidence of common mental health disorders: A systematic review and meta-analysis. Journal of Affective Disorders. Oktober 2019;257:748–57.

18. Maggio ABR, Hofer MF, Martin XE, Marchand LM, Beghetti M, Farpour-Lambert NJ. Reduced physical activity level and cardiorespiratory fitness in children with chronic diseases. Eur J Pediatr. Oktober 2010;169(10):1187–93.

19. Duncan MJ, Murphy L, Oftedal S, Fenwick MJ, Vincent GE, Fenton S. The associations between physical activity, sedentary behaviour, and sleep with mortality and incident cardiovascular disease, cancer, diabetes and mental health in adults: a systematic review and meta-analysis of prospective cohort studies. JASSB. 4. September 2023;2(1):19.

20. Grgic J, Dumuid D, Bengoechea EG, Shrestha N, Bauman A, Olds T, u. a. Health outcomes associated with reallocations of time between sleep, sedentary behaviour, and physical activity: a systematic scoping review of isotemporal substitution studies. Int J Behav Nutr Phys Act. Dezember 2018;15(1):69.

21. Clarke AE, Janssen I. A compositional analysis of time spent in sleep, sedentary behaviour and physical activity with all-cause mortality risk. The international journal of behavioral nutrition and physical activity. 2021;18(1):25.

22. Ekegren CL, Beck B, Climie RE, Owen N, Dunstan DW, Gabbe BJ. Physical Activity and Sedentary Behavior Subsequent to Serious Orthopedic Injury: A Systematic Review. Archives of Physical Medicine and Rehabilitation. Januar 2018;99(1):164-177.e6.

23. Cohen S, Doyle WJ, Alper CM, Janicki-Deverts D, Turner RB. Sleep Habits and Susceptibility to the Common Cold. Archives of Internal Medicine. 12. Januar 2009;169(1):62–7.

24. Stubbs B, Vancampfort D, Firth J, Schuch FB, Hallgren M, Smith L, u. a. Relationship between sedentary behavior and depression: A mediation analysis of influential factors across the lifespan among 42,469 people in low- and middle-income countries. Journal of Affective Disorders. März 2018;229:231–8.
